# Transcriptome analysis uncovers the diagnostic value of miR-192-5p/HNF1A-AS1/VIL1 panel in cervical adenocarcinoma

**DOI:** 10.1038/s41598-020-73523-0

**Published:** 2020-10-06

**Authors:** Junfen Xu, Jian Zou, Luyao Wu, Weiguo Lu

**Affiliations:** 1 Department of Gynecologic Oncology, Women’s Hospital, Zhejiang University School of Medicine, Hangzhou, 310006 Zhejiang China; 2 Center of Uterine Cancer Diagnosis & Therapy of Zhejiang Province, Hangzhou, 310006 Zhejiang China

**Keywords:** Gynaecological cancer, Tumour biomarkers

## Abstract

Despite the fact that the incidence of cervical squamous cell carcinoma has decreased, there is an increase in the incidence of cervical adenocarcinoma. However, our knowledge on cervical adenocarcinoma is largely unclear. Transcriptome sequencing was conducted to compare 4 cervical adenocarcinoma tissue samples with 4 normal cervical tissue samples. mRNA, lncRNA, and miRNA signatures were identified to discriminate cervical adenocarcinoma from normal cervix. The expression of VIL1, HNF1A-AS1, MIR194-2HG, SSTR5-AS1, miR-192-5p, and miR-194-5p in adenocarcinoma were statistically significantly higher than that in normal control samples. The Receiver Operating Characteristic (ROC) curve analysis indicated that combination of miR-192-5p, HNF1A-AS1, and VIL1 yielded a better performance (AUC = 0.911) than any single molecule -and could serve as potential biomarkers for cervical adenocarcinoma. Of note, the combination model also gave better performance than TCT test for cervical adenocarcinoma diagnosis. However, there was no correlation between miR-192-5p or HNF1A-AS1 and HPV16/18 E6 or E7. VIL1 was weakly correlated with HPV18 E7 expression. In summary, our study has identified miR-192-5p/HNF1A-AS1/VIL1 panel that accurately discriminates adenocarcinoma from normal cervix. Detection of this panel may provide considerable clinical value in the diagnosis of cervical adenocarcinoma.

## Introduction

Cervical cancer ranks fourth for both cancer incidence and mortality in women worldwide^1^. The predominant cases are cervical squamous cell carcinoma (SCC), and the second pool (about 10–25%) is cervical adenocarcinoma^2,3^. The former has been greatly prevented by cytological screening and HPV vaccination in developed countries. In contrast, a relative and absolute increasing proportion of adenocarcinoma was seen over the past decades, especially in women aged 20 to 39 years old^4–8^. Thus, it is important to investigate the pathogenesis of cervical adenocarcinoma and its related molecular alterations.

High-risk human papillomavirus (HR-HPV) is the major cause of cervical cancer, especially in SCC. However, HR-HPV infection rates vary from 60 to 85.8% in cervical adenocarcinoma^9–13^. At least 15% of cervical adenocarcinoma is not related to HPV infection. Other factors may also contribute to the development of adenocarcinoma. Moreover, it has been reported that cervical adenocarcinoma has a higher rate of metastasis and resistance to chemotherapy and radiotherapy than that of SCC^14,15^. In view of this clinical challenge, there is an urgent need to elucidate molecular mechanisms and explore new reliable prevention strategy for cervical adenocarcinoma.

Various types of biomarkers, especially RNA biomarkers, have been applied to clinical diagnosis for diseases for decades. Recently, high-throughput RNA sequencing technology facilitates the quantification measurements of RNA expression at the entire transcriptome level. These large-scale expression profiles of RNAs enable the detection of protein-coding RNAs (i.e., mRNAs) and non-coding RNAs (i.e., ncRNAs), which could be of profound value in terms of disease characterization. As the best-characterized type of RNA biomarkers, the mRNA biomarkers have successfully programmed into multi-gene panels for cancer diagnosis^16–19^. Lots of functionally important ncRNAs can also be used as biomarkers for increasing evidence of abnormal expression of ncRNAs closely associated with various cancers. For instance, a blind study of 22 different tumor types showed that microRNA (miRNA) expression pattern could accurately classify tumors according to tissue of origin^20^. It was reported that the expression of miR-27a is decreased in cervical cancer cell lines and it functions as a tumor suppressor in cervical adenocarcinoma by inhibiting TGF-βRI signaling pathway^21^. MiR-362-3p downregulation in cervical adenocarcinoma is associated with advanced clinical stage and poor prognosis via targeting minichromosome maintenance protein 5 (MCM5)^22^. In addition, long non-coding RNAs (lncRNAs) could be considered as biomarkers. For instance, lncRNA HOTAIR plays important roles in the development of cancer^23–26^. In cervical cancer, it was reported that high levels of circulating HOTAIR are correlated with tumor recurrence and short overall survival^27,28^. To date, the lncRNA profile in cervical adenocarcinoma remains largely unknown.

In this study, we comprehensively examined the mRNA and ncRNA expression profiles (including miRNAs and lncRNAs) in cervical adenocarcinoma and normal cervix by using transcriptome sequencing. We aim to identify and validate a mRNA/ncRNA-combined signature that could improve the screening or early detection of cervical adenocarcinoma. We revealed that a panel with miR-192-5p, HNF1A-AS1 and VIL1 could accurately discriminate adenocarcinoma from normal cervix.

## Methods

### Human tissue samples

**Figure 1:**
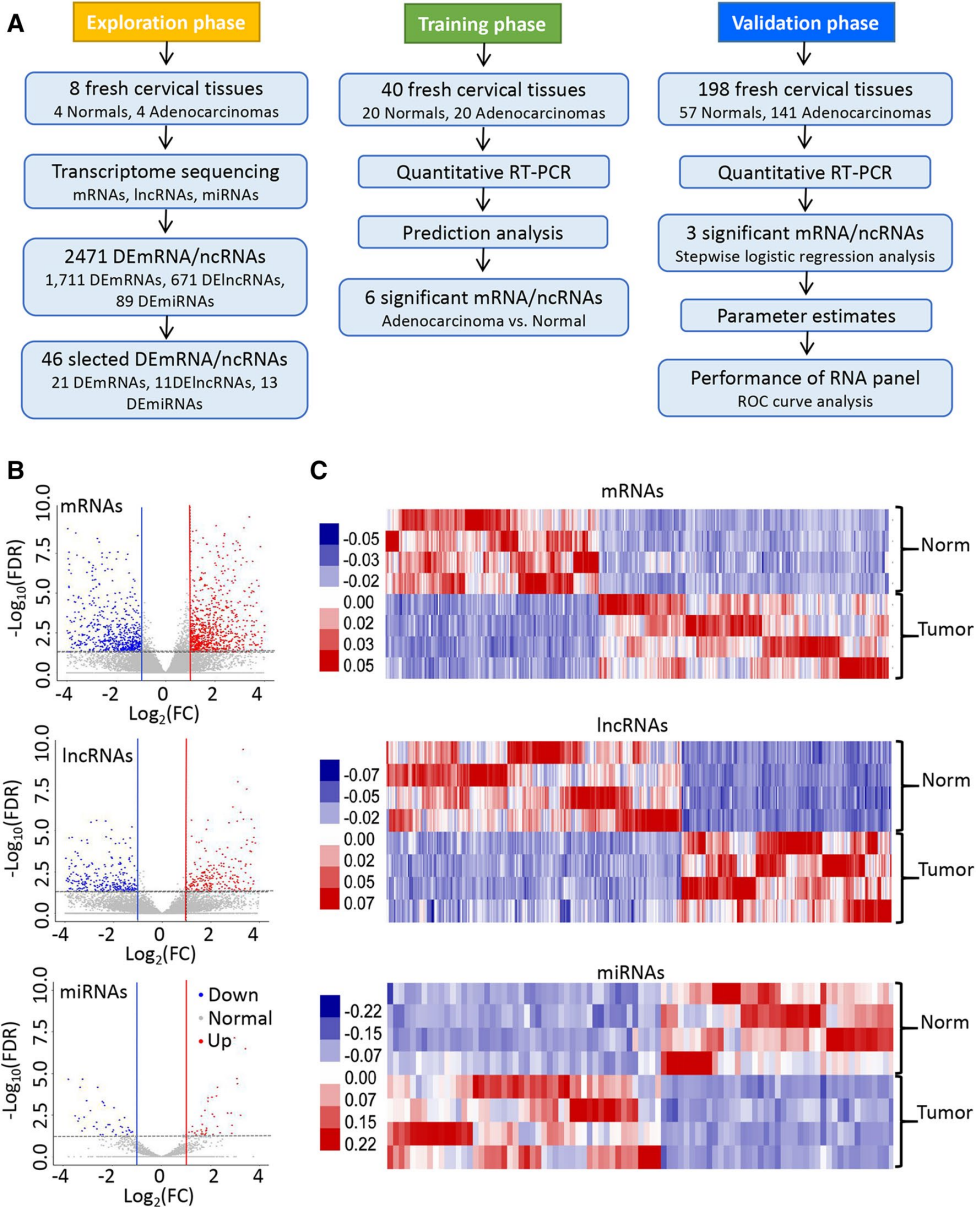
Study design and mRNA/lncRNA/miRNA profiling in human cervical adenocarcinoma and normal cervical tissues. (**A**) Flowchart illustrating the discovery of miR-192-5p/HNF1A-AS1/VIL1 panel for discriminating cervical adenocarcinoma. (**B**) Volcano plot visualization of differential mRNA, lncRNA, and miRNA expressions between 4 adenocarcinoma and 4 normal cervical tissues by transcriptome sequencing. Blue, down-regulated RNAs; Red, up-regulated RNAs; FC, fold change; FDR, false-discovery rate. (**C**) Heatmaps of differentially expressed mRNAs, lncRNAs, and miRNAs in 4 cervical adenocarcinoma and 4 normal cervical tissue samples. Red, up-regualation in cervical adenocarcinoma; Blue, down-regulation in cervical adenocarcinoma.

Three groups of cervical adenocarcinoma and normal cervical tissue samples were all collected from the Women’s Hospital, Zhejiang University School of Medicine. Cervical samples, including 4 normal and 4 adenocarcinoma tissue samples for RNA sequencing were obtained from January 2018 to February 2018. The second panel of cervical tissue samples containing 20 normal and 20 adenocarcinoma tissue samples for the first step validation by RT-qPCR analysis, were collected from March 2018 to March 2019. The third panel of cervical tissue samples including 57 normal and 141 adenocarcinoma tissue samples for the second step validation by RT-qPCR analysis, were obtained from January 2009 to December 2017. None of these patients received radiotherapy or chemotherapy before operation. Diagnosis of these samples was confirmed independently by two senior pathologists before further analysis. After removal from the patients, tissue samples were put immediately in liquid nitrogen and stored at -80 °C until use. This study was approved by the Ethics Committee of Women’s Hospital of Zhejiang University (IRB-2019062-R). All steps of the study were performed with the relevant guidelines and regulations (according to the Declaration of Helsinki Principles). Written informed consent was obtained from each patient prior to the study. The three different phases of our study design are shown in Fig. 1A and the patients’ clinical-pathological information is summarized in supplementary Table S1.

### Transcriptome sequencing

Four cervical adenocarcinoma tissues and 4 normal cervical tissues were used for transcriptome sequencing. Transcriptome Sequencing was performed by NovelBio Corp. Laboratory, Shanghai, China. Our transcriptome sequencing data have been deposited in NCBI Gene Expression Omnibus (GEO) datasets with the accession number GSE145372 (https://www.ncbi.nlm.nih.gov/geo). RNA was utilized to construct both rRNA depletion library and miRNA library. The products were sequenced on Hiseq Sequencer and HiSeq Xten platform (Illumina, San Diego, CA), respectively. The reads were then mapped to Human genome (GRCh38 NCBI) utilizing HISAT2 (-5 5 -3 5 -p 8 -min-intronlen 20 -max-intronlen 500,000 -k 3)^29^ and HTSeq was used to calculate the gene count of mRNA and lncRNA annotated by NCBI genome gff3 file^30^. To achieve miRNA expression, the reads were filtered (length ranged from 15 to 33 bp; Q20 > 20%; Trim end Q15) and mapped to Human miRNA database (miRBase v22.0) and Human genome (GRCh38 NCBI), together with mapping to the RFAM database (https://rfam.xfam.org/) for miRNA quality control by BWA (bwa aln -n 0.04 -e 3 -l 32 -k 2 -t 12)^31^. We applied DESeq2 package^32^ to discover the differentially expressed mRNA, lncRNA, and miRNA under the following criteria: Absolute Fold Change > 2 and FDR < 0.05.

For heatmap generation, the sample FPKM (Fragments per kilobases million reads) values of Different expression genes were clustered using Software Cluster 3.0 (https://bonsai.hgc.jp/~mdehoon/software/cluster/software.htm). The rows and columns expression matrix was centered according to the mean value and finally normalized to value ranged from − 1 to 1. Average linkage was applied to cluster genes and samples with the uncentered correlation method. The heatmap was finally visualize by TreeView (https://bonsai.hgc.jp/~mdehoon/software/cluster/software.htm).

The interactions among lncRNAs, miRNAs and mRNAs were predicted using the miRNA target prediction software miranda (-strict -sc 150 -en -20)^33^, RNAhybrid (-e -25 -b 1 -f 2,7 -m 2,000,000 -s 3utr_human -n 50)^34^ for non-coding RNA target and targetscan for mRNA target^35^. An mRNA-miRNA-lncRNA network was constructed according to the consistent target miRNAs of the lncRNAs and mRNAs. Co-expression network was constructed according to the normalized expression values of genes selected from differentially expressed mRNAs^36^. For each pair of genes, we calculated the Pearson’s correlation, chose the significant correlation pairs (FDR < 0.05) and then construct the network^37^.

### Quantitative reverse-transcriptase PCR (RT-qPCR) assay

Total RNA was isolated from normal cervix and cervical adenocarcinoma tissues with Trizol reagent (Life Technologies, Carlsbad, USA) according to the manufacturer’s instructions. cDNA for mRNA and lncRNA was synthesized by PrimeScript RT reagent kit with gDNA Eraser (TaKaRa Biomedical Technology Co., Ltd). For miRNA, RNA was reverse-transcribed into cDNA by the PrimeScript RT reagent kit (TaKaRa). RNA expression was quantified by qPCR on a StepOne Plus Real-Time PCR System (Applied Biosystems; Thermo Fisher Scientific, Inc) using TB Green Premix Ex Taq kit (TaKaRa). GAPDH or U6 were used as controls. Relative RNA expression levels were analyzed using the 2^−ΔΔCT^ method. Specific EzOmics miRNA qPCR detection primer sets were bought from Biomics Biotech, Nantong, China. The specific primers of mRNA and lncRNA were listed in Appendix Table S2.

### ROC curve analysis

Receiver operating characteristic (ROC) analysis was performed as previously described^38^. Briefly, the qPCR RNA expression validation results (2^−ΔΔCT^) determined for 5 candidates, including VIL1, HNF1A-AS1, MIR194-2HG, SSTR5-AS1, miR-192-5p, and miR-194-5p, in 57 normal cervix and 141 cervical adenocarcinoma tissue samples were uploaded to IBM SPSS 24.0 and applied for ROC curve analysis. The area under the ROC curve (AUC) is interpreted as the average value of sensitivity (true-positive fraction) for all possible values of specificity (false-positive fraction). AUC < 0.5, not significant; 0.5 < AUC < 0.7, low significant; 0.7 < AUC < 1.0, high significance. ROC curve and logistic regression analysis were also used to assess the performance of combined RNA detection in discriminating cervical adenocarcinoma.

### Statistical analysis

We statistically analyzed the data using Student’s *t* test or Mann–Whitney U-test as appropriate with SPSS 24.0 statistical software (SPSS, Chicago, IL, USA). The results of our experiments are shown as mean value ± standard deviation. Correlations were calculated by Spearman rank correlation analysis. *P*-values < 0.05 was considered statistically significant.

## Results

### Identification of mRNA, lncRNA, and miRNA expression signatures in cervical adenocarcinoma

We conducted transcriptome sequencing to characterize the expression profiles of mRNAs, lncRNAs, and miRNAs in 4 human cervical adenocarcinoma tissues and 4 normal cervical tissues. Using the same stringent criteria, a total of 1,711 differentially expressed mRNAs (DEmRNAs), 671 DElncRNAs, and 89 DEmiRNAs were identified from cervical adenocarcinoma compared with normal tissue samples. As shown in Fig. 1B, volcano plots were generated to display the distribution of the DEmRNAs, DElncRNAs, and DEmiRNAs. The heatmap of each DERNA group showed clear separation and consistency in the expression profiles of the cervical adenocarcinoma and normal samples (Fig. 1C).

### Validation of the differentially expressed RNAs in clinical cervical tissue samples

**Figure 2:**
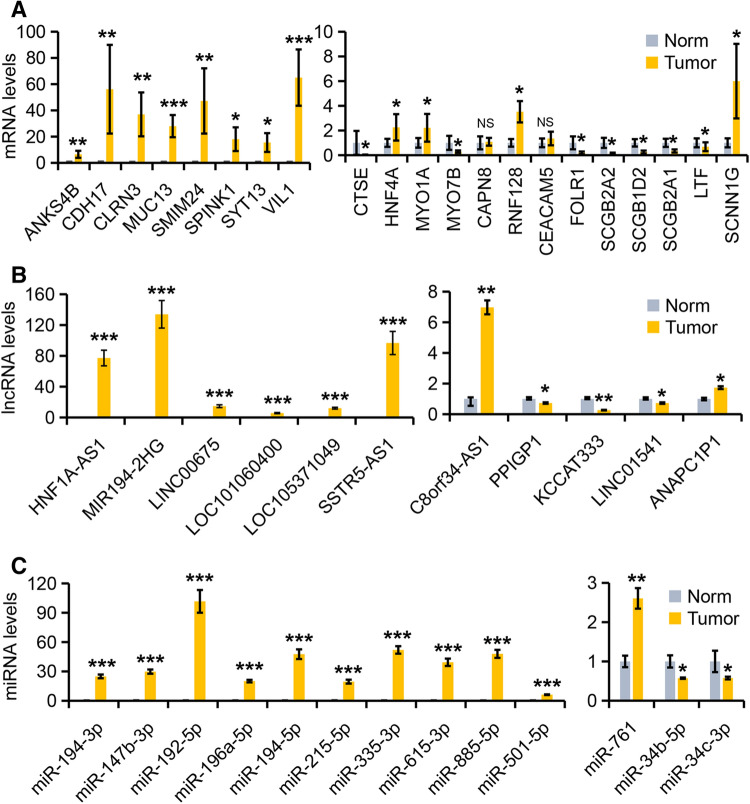
Experimental validation of selected mRNAs, lncRNAs, and miRNAs in cervical tissue samples. (**A**–**C**) TB Green RT-qPCR validation of 21 DEmRNAs (**A**), 11 DElncRNAs (**B**), and 13 DEmiRNAs (**C**) in 40 clinical cervical tissue samples containing 20 normal (Norm) and 20 adenocarcinoma (Tumor) tissue samples. Data of the expression levels were shown as mean (2^−ΔΔCT^) ± SD.

21 DEmRNAs (ANKS4B, CDH17, CLRN3, MUC13, SMIM24, SPINK1, SYT13, VIL1, CTSE, HNF4A, MYO1A, MYO7B, CAPN8, RNF128, CEACAM5, FOLR1, SCGB2A2, SCGB1D2, SCGB2A1, LTF, and SCNN1G), 11 DElncRNAs (HNF1A-AS1, MIR194-2HG, LINC00675, LOC101060400, LOC105371049, SSTR5-AS1, C8orf34-AS1, PPIGP1, KCCAT333, LINC01541, and ANAPC1P1), and 13 DEmiRNAs (miR-194-3p, miR-147b-3p, miR-192-5p, miR-196a-5p, miR-194-5p, miR-215-5p, miR-335-3p, miR-615-3p, miR-885-5p, miR-501-5p, miR-761, miR-34b-5p, and miR-34c-3p) generated by transcriptome sequencing analysis as described above were selected based on their high abundance and large fold changes in expression for further validation by RT-qPCR with an independent cohort of 40 cervical tissue samples containing 20 normal and 20 adenocarcinoma tissues (Fig. 2A–C).

Of the 21 significantly changed mRNAs, the expression levels of 14 mRNAs (ANKS4B, CDH17, CLRN3, MUC13, SMIM24, SYT13, VIL1, CTSE, HNF4A, MYO1A, MYO7B, RNF128, SCGB2A1, and SCNN1G) were newly found dysregulated in cervical adenocarcinoma in this study. Of the 11 significant lncRNAs, only LINC00675 and HNF1A-AS1 have been reported in cervical cancer cells and the left 9 lncRNAs (MIR194-2HG, LINC00675, LOC101060400, LOC105371049, SSTR5-AS1, C8orf34-AS1, PPIGP1, KCCAT333, LINC01541, and ANAPC1P1) were newly identified in this study. Of the 13 significant miRNAs, 7 miRNAs has been reported in association with cervical cancer and the rest 6 miRNAs (miR-194-3p, miR-147b-3p, miR-335-3p, miR-615-3p, miR-501-5p, and miR-761) were newly identified in this study.

Our RT-qPCR studies confirmed that the expression levels of 17 mRNAs showed the similar expression trends with RNA-seq data. Among them, the expression levels of 12 mRNAs ( ANKS4B, CDH17, CLRN3, MUC13, SMIM24, SPINK1, SYT13, VIL1, HNF4A, MYO1A, RNF128, and CEACAM5) were significantly increased and the ones of 5 mRNAs (FOLR1, SCGB2A2, SCGB1D2, SCGB2A1, and LTF) were significantly decreased in 20 cervical adenocarcinoma tissues over the 20 normal cervical tissues (Fig. 2A). For lncRNAs, we found that 9 lncRNAs showed the similar expression trends with RNA-seq results. Among them, the expression levels of 6 lncRNAs (HNF1A-AS1, MIR194-2HG, LINC00675, LOC101060400, LOC105371049, and SSTR5-AS1) were significantly increased while the levels of the left 3 lncRNAs (PPIGP1, KCCAT333, and LINC01541) were significantly decreased using the same tissue samples as in Fig. 2A,B. For miRNA expression validation, we confirmed that all these 13 miRNAs expressed similar trends with RNA-seq results. Among them, the expression levels of 11 miRNAs (miR-194-3p, miR-147b-3p, miR-192-5p, miR-196a-5p, miR-194-5p, miR-215-5p, miR-335-3p, miR-615-3p, miR-885-5p, miR-501-5p, and miR-761) were significantly increased while the levels of the left 2 miRNAs (miR-34b-5p and miR-34c-3p) were significantly decreased in the above same cervical adenocarcinoma tissue group over the normal cervical tissue group (Fig. 2C).

Combined with the validation results, we finally chose VIL1, HNF1A-AS1, MIR194-2HG, SSTR5-AS1, miR-192-5p, and miR-194-5p as the most promising candidates for further investigation because of their large fold changes in expression and relatively little variation in each group.

### Diagnostic values of potential biomarkers in cervical adenocarcinoma

**Figure 3:**
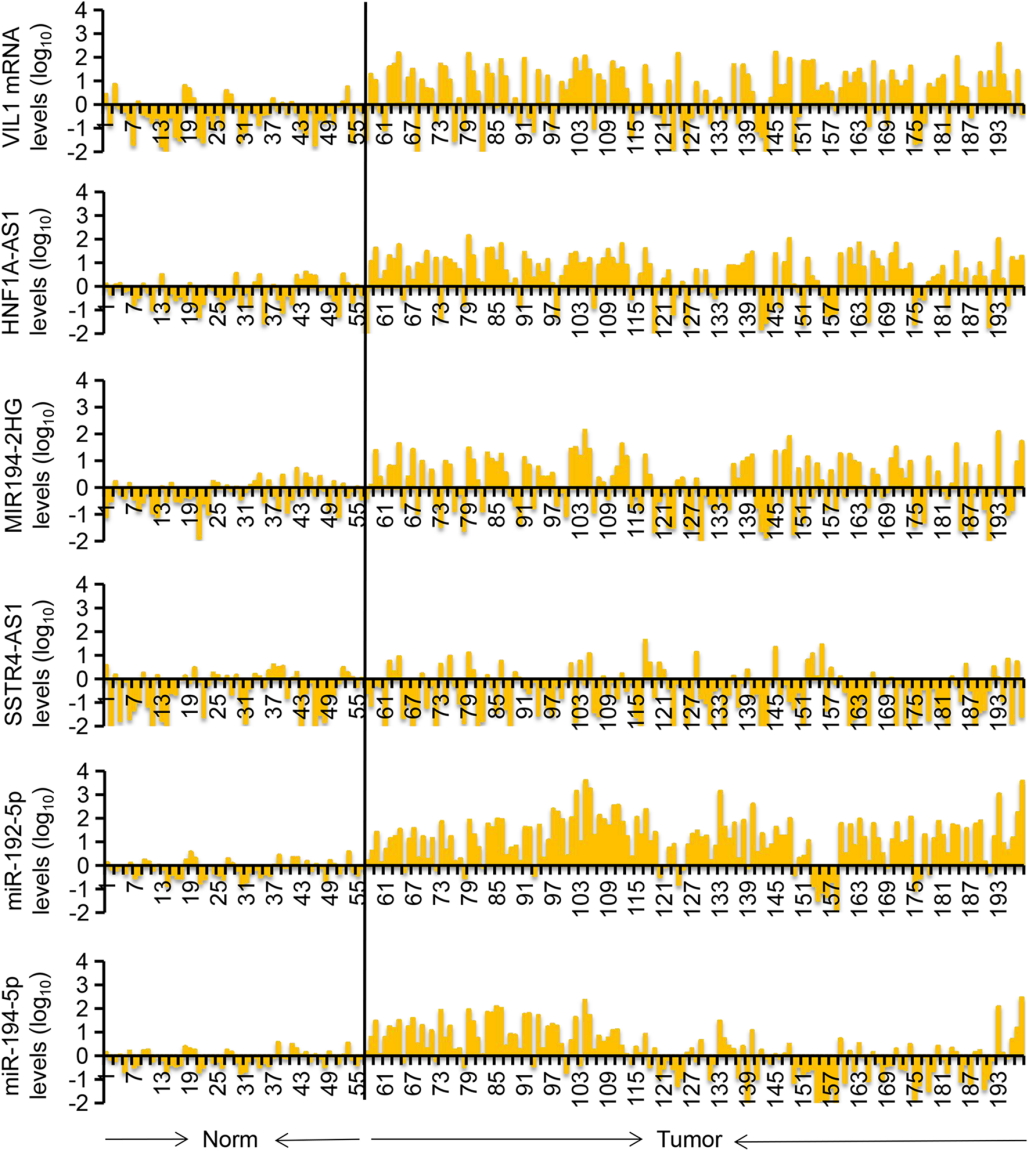
Characterization of VIL1, HNF1A-AS1, MIR194-2HG, SSTR5-AS1, miR-192-5p, and miR-194-5p expression pattern in another cohort of 198 human cervical tissue samples. The TB Green RT-qPCR validation confirmed the distribution of expression levels of VIL1, HNF1A-AS1, MIR194-2HG, SSTR5-AS1, miR-192-5p, and miR-194-5p in another 57 normal cervical and 141 adenocarcinoma tissue samples. Data are shown as log_10_ (fold changes of cervical adenocarcinoma versus average normal samples).

The expression of VIL1 mRNA, HNF1A-AS1, MIR194-2HG, SSTR5-AS1, miR-192-5p, and miR-194-5p were further investigated by RT-qPCR in another 57 normal cervical tissues and 141 adenocarcinoma tissues-. Similarly, all these 5 candidates were abnormally expressed in cervical adenocarcinoma (Fig. 3).

**Figure 4:**
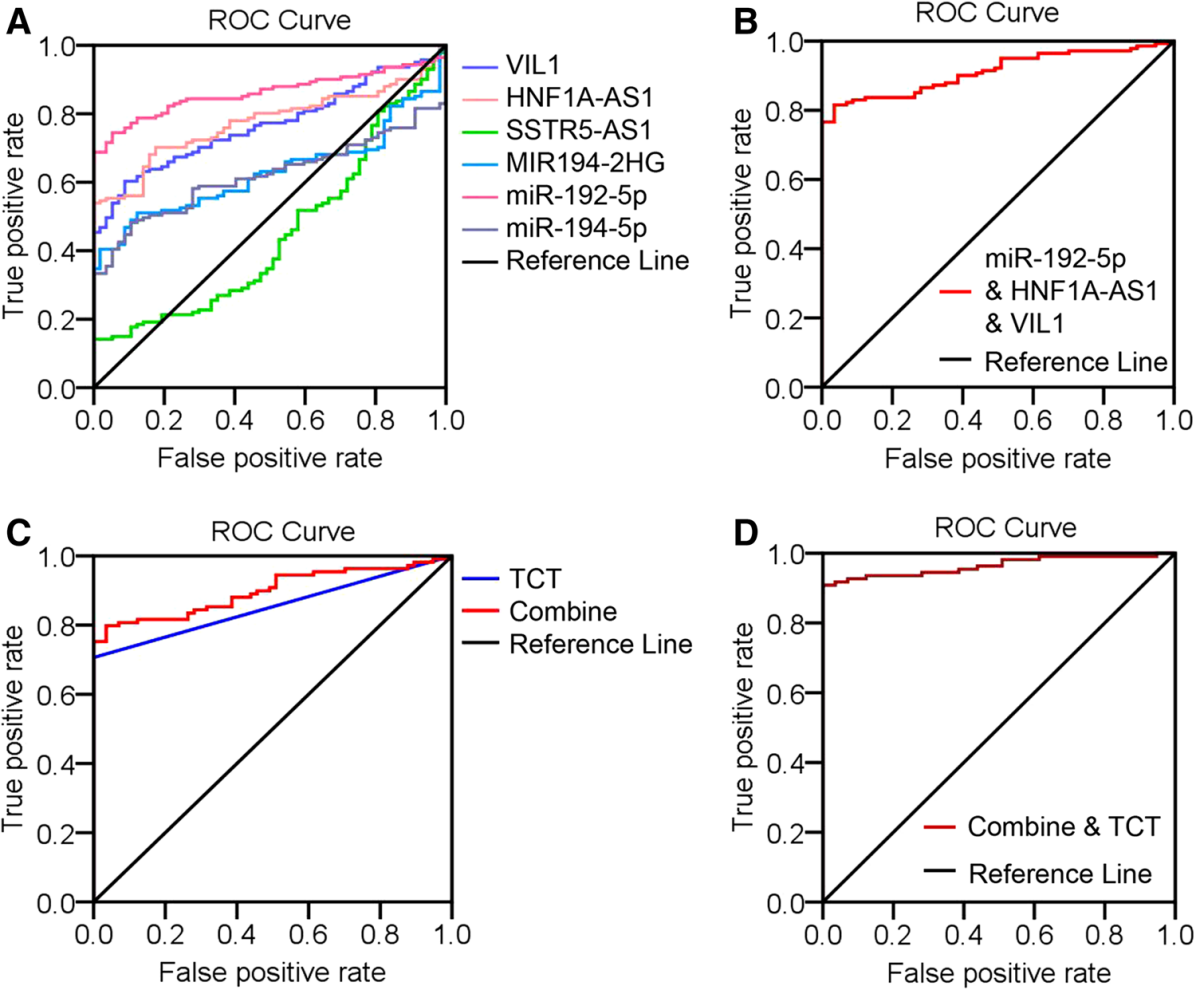
ROC curve analysis of the altered mRNA, lncRNA, and miRNA candidates in normal cervical and adenocarcinoma tissues. (**A**) ROC analysis of single VIL1, HNF1A-AS1, MIR194-2HG, SSTR5-AS1, miR-192-5p, and miR-194-5p detection for cervical adenocarcinoma diagnosis. (**B**) ROC analysis of the logit model with miR-192-5p/HNF1A-AS1/VIL1 panel on 57 normal cervical and 141 adenocarcinoma tissue samples. (**C**) ROC analysis of miR-192-5p/HNF1A-AS1/VIL1 panel and TCT test in 166 patients who have received TCT tests. (**D**) ROC analysis of combination of the miR-192-5p/HNF1A-AS1/VIL1 panel and TCT tests on 57 normal cervical and 109 adenocarcinoma tissue samples.

To determine their diagnostic values in cervical adenocarcinoma, we compared the levels of sensitivity and specificity using receiver operating characteristic (ROC) analysis. The generated RT-qPCR results were analyzed by ROC analysis according to comparisons of normal versus cervical adenocarcinoma samples. As shown in Fig. 4A and Table S2, the area under the curve (AUC) to discriminate cervical adenocarcinoma from normal cervix was 0.767 (95% CI 0.704–0.831) for VIL1, 0.774 (95% CI 0.711–0.837) for HNFA1-AS1, 0.629 (95% CI 0.554–0.703) for MIR194-2HG, 0.449 (95% CI 0.362–0.535) for SSTR5-AS1, 0.862 (95% CI 0.811–0.912) for miR-192-5p, and 0.619 (95% CI 0.544–0.694) for miR-194-5p. Using the AUC value of > 0.70 as a cutoff for high significance, we identified VIL1, HNF1A-AS1, and miR-192-5p as specific biomarkers for the cervical adenocarcinoma group, suggesting the valuable diagnostic potential of these 3 candidates in cervical adenocarcinoma patients.

We then applied stepwise logistic regression to the 198 cervical samples starting from the 3 candidates (miR-192-5p, HNF1A-AS1, and VIL1) and built a logit model logit (p) = − 1.546 + 0.488 * (miR-192-5p) + 0.265 * (HNF1A-AS1) + 0.154 * (VIL1). Applying this model to the same 198 cervical samples yields an AUC of 0.911 (95% CI 0.872–0.950) (Table S3). This combined miR-192-5p/HNF1A-AS1/VIL1 panel achieved greater AUC value than any single candidate detection, indicating a promising biomarker pattern for discriminating cervical adenocarcinoma from normal cervix. The corresponding ROC curve is shown in Fig. 4B.

Of the 141 cervical adenocarcinoma patients, 109 patients have received thinprep cytologic test (TCT) for screening cervical cancer before cervical tissue biopsies, 24 patients directly conducted colposcopically directed tissue biopsies because of the large visible cervical tumors, and the rest 8 patients only have received HPV detection followed by colposcopically directed tissue biopsies. For all of the 57 healthy control samples, TCT tests were normal and HPV tests were negative. Among the 109 patients, 77 patients were presented with abnormal TCT tests (ASCUS/+) and 32 presented with normal TCT tests. We then plotted ROC curves for miR-192-5p/HNF1A-AS1/VIL1 panel as well as TCT tests in the 57 normal control group and 109 adenocarcinoma group. As shown in Fig. 4C, miR-192-5p/HNF1A-AS1/VIL1 panel detection showed advantages over the TCT tests with greater AUC (0.898 vs. 0.853) (Table S3). Further logistic regression analysis identified combination of miR-192-5p/HNF1A-AS1/VIL1 panel and TCT tests to achieve greater AUC (0.964) than the TCT test or miR-192-5p/HNF1A-AS1/VIL1 panel detection (Fig. 4D, Table S4), suggesting a potential biomarker for cervical adenocarcinoma.

### Putative target and co-expression analysis for miR-192-5p, HNF1A-AS1, and VIL1

**Figure 5:**
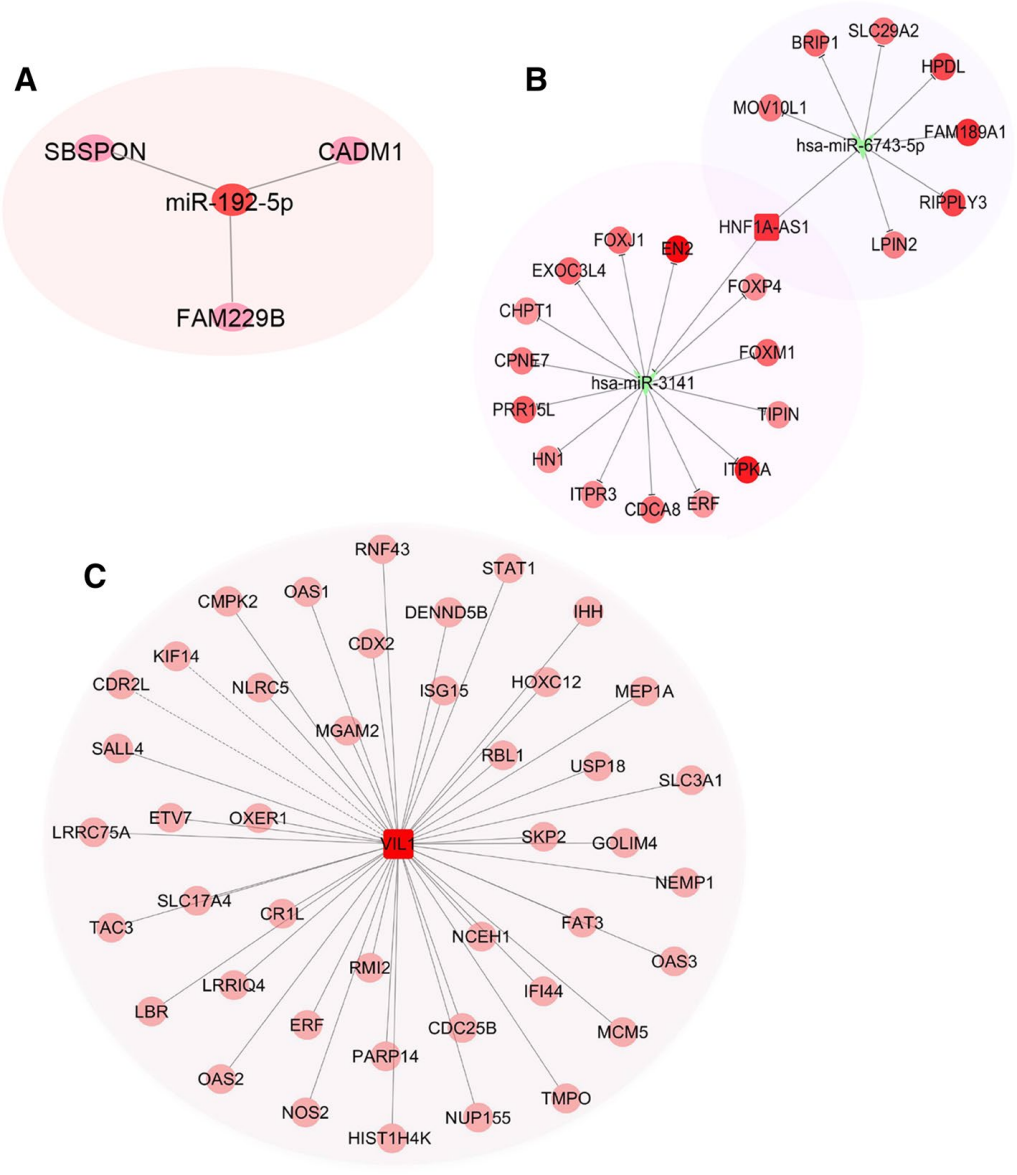
Predicted regulatory networks of miR-192-5p, HNF1A-AS1, and VIL1. (**A**) Expression relationship between miR-192-5p and target mRNAs. Putative miR-192-5p targets were predicted by targetscan and miranda. The genes which were found down-regulated in cervical adenocarcinoma tissue samples from the transcriptome sequencing results were shown. (**B**) HNF1A-AS1-miRNA-mRNA competing endogenous RNA networks were shown. Green, down-regulated miRNAs; Red, up-regulated mRNAs. The brightness of the mRNA circle represent the degree of the connection. (**C**) Co-expression network of VIL1 generated by Cytoscape. The genes which were also up-regulated in cervical adenocarcinoma tissues samples were shown.

Biological enrichment analysis was also performed to further analyze the putative biological functions of miR-192-5p, HNF1A-AS1, and VIL1. MiRNA-mRNA interactions by miranda and targetscan analysis showed that the downregulated expression of CADM1, SBSPON, and FAM229B in cervical adenocarcinoma samples might be the potential target genes for miR-192-5p (Fig. 5A). For HNF1A-AS1, an integrated lncRNA-miRNA-mRNA network was conducted. The ceRNA analysis showed that HNF1A-AS1 functioned as a sponge of miR-3141 and miR-6743-5p and regulated a set of miRNA target genes (Fig. 5B). The co-expression analysis showed that VIL1 was co-expressed with multiple genes, which are also dysregulated in cervical adenocarcinoma (Fig. 5C).

### The correlation between miR-192-5p/HNF1A-AS1/VIL1 panel and HPV oncogenes

**Figure 6:**
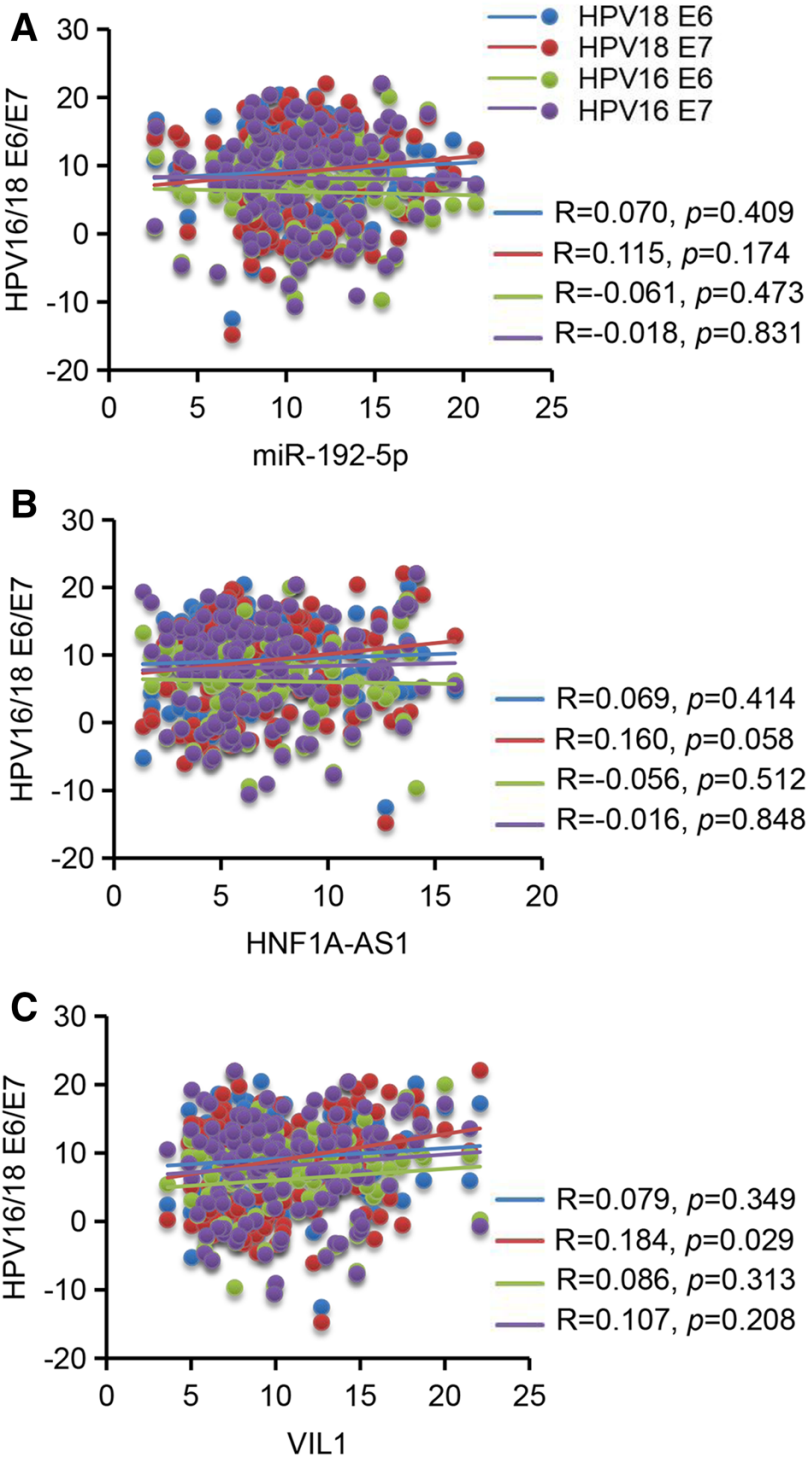
Correlation between miR-192-5p/HNF1A-AS1/VIL1 panel and HPV oncogenes. (**A**) The association between miR-192-5p and HPV16E6, HPV E7, HPV18 E6, and HPV18 E7 was analyzed by Spearman rank correlation , respectively. (**B**) The relationship between HNF1A-AS1 and HPV16E6, HPV E7, HPV18 E6, and HPV18 E7 was shown. (**C**) The expression of VIL1 was positively correlated with HPV18 E7, but not HPV16 E6, HPV16 E7, or HPV18 E6. Data of the expression levels for correlation analysis were shown as ΔCT. GAPDH was used for normalization.

The most common HPV types distributed in cervical adenocarcinoma were HPV16 and HPV18^39^. Expression of HPV oncogenes E6 and E7 is a key event in the malignant transformation of HPV-infected cervical cells^40^. To determine whether the increased levels of miR-192-5p, HNF1A-AS1, and VIL1 expressions were associated with viral E6 or E7, we tested the expression of HPV16 E6, HPV16 E7, HPV18 E6, and HPV18 E7 mRNAs in the above 141 cervical adenocarcinoma tissue samples. The expression of miR-192-5p was not significantly correlated with HPV16 E6, HPV16 E7, HPV18 E6, or HPV18 E7 (Fig. 6A). Also, there was no positive correlation between the expression of HNF1A-AS1 and HPV16/18 E6/E7 (Fig. 6B). For VIL1, it was significantly positive correlated with HPV18 E7, but not HPV18 E6, HPV16 E6, or HPV16 E7 (Fig. 6C). However, the correlation was very weak (R = 0.184, *P* = 0.029). These results indicate that the altered expression of miR-192-5p and HNF1A-AS1 was not attributed to viral E6 or E7, and the expression of VIL1 might be attributed to HPV18 E7 expression.

## Discussion

Cervical adenocarcinoma is one of the main causes for cervical cancer-related deaths, and is a heterogeneous disease with multiple molecular dysregulation. Finding of specific diagnostic biomarkers may conceal the important signature of this malignancy. Current mainstream biomarkers involved in cervical cancer are from single type of RNA markers. For instance, Tian et al. has identified miR-424/375/218 and miR-424/375 as two multimarker panels for discriminating cervical intraepithelial neoplasia (CIN) 2+ and CIN3+ , respectively^41^. It is not excluded that combination of different RNA types is more valuable as effective diagnosis biomarkers for cervical adenocarcinoma. In this study, we identified distinct mRNAs, lncRNAs, and miRNAs to gain insights into the molecular events associated with cervical adenocarcinoma. Accordingly, a panel of mRNA/ncRNA model has been built to discriminate cervical adenocarcinoma from normal tissues.

First, we identified mRNA, lncRNA and miRNA signatures in cervical adenocarcinoma tissues by transcriptome sequencing that enabled us to efficiently find potential novel RNA targets for diagnosis. Second, we assessed the transcriptome sequencing data by choosing 46 significantly altered candidates, including 21mRNAs, 11lncRNAs and 13 miRNAs, to derive reliable RNA markers. Third, we examined the expression signatures of the 6 putative mRNAs/ncRNAs in another independent cohort of cervical adenocarcinoma tissues and identified a panel containing miR-192-5p, HNF1A-AS1 and VIL1 as the effective biomarker set for cervical adenocarcinoma. Finally, we revealed that the combination of miR-192-5p/HNF1A-AS1/VIL1 panel and TCT test achieves better AUC than any single detection, providing important clues for clinical implementation.

HR-HPV infection plays important roles in the etiology of cervical adenocarcinoma. The continuous expression of viral oncogenes E6 and E7 is critical in the malignant transformation. Our identification of miR-192-5p/HNF1A-AS1/VIL1 panel offers an exciting novel insight into the development of cervical adenocarcinoma. We further compared these panel markers with HPV16 or HPV18 E6 or E7. We found VIL1 was positively correlated with HPV18 E7. However, no significant correlation was observed between miR-192-5p or HNF1A-AS2 with HPV16 or HPV18 E6 or E7 in cervical adenocarcinoma tissues. Our results indicate that the alteration of non-HPV-driven host molecules may also play important roles in the pathogenesis of cervical adenocarcinoma.

Aberrant expression of miR-192-5p has been reported in several tumors, such as hepatocellular carcinoma (HCC), non-small cell lung cancer (NSCLC), pancreatic cancer, and cervical cancer^42–45^. It was reported that miR-192-5p expression was significantly decreased in HCC, especially in cancer stem cell (CSC) + HCC. miR-192-5p could functionally suppressed CSC features in HCC cells through targeting PABPC4^44^. Differently, the levels of serum miR-192-5p was significantly upregulated in nasopharyngeal carcinoma and cervical cancer^43,46^. This controversy indicate that miR-192-5p acts as either tumor-suppressive or oncogenic miRNA in different tumor types. Our results have shown a significant increase of miR-192-5p expression in cervical adenocarcinoma tissues compared with the normal group, suggesting miR-192-5p as an important oncogene in cervical adenocarcinoma. Strikingly, miR-192-5p alone yielded an excellent AUC in discriminating adenocarcinoma from the healthy controls, which further points out the potential applicable value of miR-192-5p as a diagnostic marker for cervical adenocarcinoma. The potential predicted targets of miR-192-5p include CADM1, SBSPON and FAM229B, which were down-regulated in adenocarcinoma from our transcriptome sequencing data. To test whether miR-192-5p expression is regulated by HPV that contributes to the cervical tumorigenesis, we analyzed the correlation between miR-192-5p and HPV16 or 18 E6 or E7. However, the altered expression of miR-192-5p was not correlated with viral E6 or E7 expression and further studies are needed to address the roles of miR-192-5p in cervical adenocarcinoma.

Among the cancer-related lncRNAs, lncRNA HNF1A antisense RNA 1 (HNF1A-AS1) has been reported to be highly expressed and act as an oncogene in various human malignancies^47–49^. Studies have demonstrated that HNF1A-AS1 participates in various cellular processes, including proliferation, apoptosis, autophagy, migration, and invasion, and thus promotes tumorigenesis and cancer progression. For instance, HNF1A-AS1 promotes hepatocellular carcinoma cell proliferation by repressing the NKD1 and p21 expression via interacting with EZH2, or by sponging miR-30b-5p to promote autophagy^50,51^. HNF1A-AS1 also promotes tumor cell proliferation and metastasis in a Wnt/β-catenin-dependent manner in osteosarcoma and colorectal cancer^52,53^. However, its clinical significance has not been elucidated in cervical adenocarcinoma. In this study, we have shown that the expression levels of HNF1A-AS1 are markedly upregulated in cervical adenocarcinoma tissue samples compared to normal cervical tissue samples. ROC curve analysis showed that HNFA1-AS1 could discriminate adenocarcinoma from normal cervix with an AUC of 0.774. ceRNA analysis showed that HNF1A-AS1 may act as a sponge of miR-3141 and miR-6743-5p and regulate the downstream targets of these two miRNAs.

Villin 1 (VIL1) is a calcium-regulated, actin-binding protein that is associated with the microfilament bundles of brush border microvilli. Under normal physiological conditions, VIL1 is expressed in epithelial cells of gastrointestinal and urogenital tracts. Recently, VIL1 has been reported to be overexpressed in several tumors including gastrointestinal neuroendocrine tumor^54^, lung cancer^55^, hepatocellular carcinoma^56^, colon cancer^57^, and endometrial adenocarcinoma^58^. Nagashio et al. showed that VIL1 was specifically expressed in adenocarcinoma and large cell neuroendocrine carcinoma in sera from pulmonary carcinoma patients, suggesting VIL1 as a useful marker to distinguish adenocarcinoma/large cell neuroendocrine carcinoma from squamous cell carcinoma/small cell carcinoma in lung cancer^55^. Nakamura et al. also reported VIL1 as a potential diagnostic marker for cervical adenocarcinoma with poor radioresponse by immunohistochemical analysis. Consistent with other reports, we observed significant upregulation of VIL1 mRNAs in human cervical adenocarcinoma tissues. Wang et al. reported that VIL1 functions as an anti-apoptotic protein via maintaining mitochondrial integrity^59^. The anti-apoptotic function can also be induced by either inhibition of the caspase-9 and caspase-3 or activation of the pro-survival proteins, phosphatidylinositol 3-kinase and phosphorylated Akt. The results of co-expression analysis shows that VIL1 is co-expressed with many genes up-regulated in cervical adenocarcinoma. Moreover, we found the expression of VIL1 was positively correlated with HPV18 E7. Thus, we propose that VIL1 might be a candidate of HPV18-related oncogene in cervical adenocarcinoma. Further functional studies are required to verify this possibility.

Although above findings of this study show important clinical implications for diagnosis of cervical adenocarcinoma, our study has some limitations. Firstly, our results were derived from a clinic-based patients instead of a general population. Also, the TCT tests were not detected in all of the cervical adenocarcinoma patients and it may possess biases. Thus, future studies are needed to confirm the role of the multimarker panel in a general population.

## Conclusion

In summary, we have determined specific mRNA, lncRNA, and miRNA signatures between cervical adenocarcinoma and normal cervix and identified a novel panel consisting of miR-192-5p, HNF1A-AS1 and VIL1 for discriminating cervical adenocarcinoma form normal tissues. This panel has promising clinical value in the early diagnosis of cervical adenocarcinoma.

## Supplementary information

Supplementary information.

## Abbreviations

SCC: Cervical squamous cell carcinoma
NcRNA: Non-coding RNA
MiRNA: MicroRNA
LncRNA: Long non-coding RNA
ROC: Receiver operating characteristic
AUC: Area under the ROC curve
RT-qPCR: Quantitative reverse-transcriptase PCR
HNF1A-AS1: LncRNA HNF1A antisense RNA 1
VIL1: Villin 1
