# Supplementary material for: Transcriptome analysis uncovers the diagnostic value of miR-192-5p/HNF1A-AS1/VIL1 panel in cervical adenocarcinoma
Source: Sci Rep. 2020 Oct 6;10:16584. doi: 10.1038/s41598-020-73523-0 (PMC7538942; doi:10.1038/s41598-020-73523-0)
Supplement: Supplementary file 1 — Supplementary information. [file 41598_2020_73523_MOESM1_ESM.pdf]

Transcriptome analysis uncovers the diagnostic value of miR-192-5p / HNF1A-AS1 / VIL1 panel in cervical adenocarcinoma

Junfen Xu [1,*], Jian Zou [1], Luyao Wu [1], Weiguo Lu [1,2,*]

[1] Department of Gynecologic Oncology, Women's Hospital, Zhejiang University School of Medicine, Hangzhou, China

[2] Center of Uterine Cancer Diagnosis & Therapy of Zhejiang Province

[*] Co-corresponding authors

Corresponding author 1:

Weiguo Lu, M.D., PhD., Women's Hospital, Zhejiang University School of Medicine, Hangzhou, Zhejiang 310006, China, Tel.: +86 571 87031130, Fax: +86 571 87061878, Email: lbwg@zju.edu.cn

Corresponding authors 2:

Junfen Xu, M.D., Women's Hospital, Zhejiang University School of Medicine, Hangzhou, Zhejiang 310006, China, Tel.: +86 571 89992145, Fax: +86 571 87061878, Email: xjfzu@zju.edu.cn

Table S1. Patients' information in this study

Patient No.	Age	FIGO stage[a]	Histological grade[b]	Lymph node metastasis[c]
Exploration phase				
Norm1	45	/	/	/
Norm2	49	/	/	/
Norm3	48	/	/	/
Norm4	46	/	/	/
Tumor1	49	IB1	2	0
Tumor2	47	IB1	1	0
Tumor3	40	IIA1	2	0
Tumor4	46	IIA1	2	0
Training phase				
Norm5	34	/	/	/
Norm6	55	/	/	/
Norm7	43	/	/	/
Norm8	46	/	/	/
Norm9	37	/	/	/
Norm10	38	/	/	/
Norm11	47	/	/	/
Norm12	41	/	/	/
Norm13	44	/	/	/
Norm14	49	/	/	/
Norm15	42	/	/	/
Norm16	36	/	/	/
Norm17	31	/	/	/
Norm18	55	/	/	/
Norm19	53	/	/	/
Norm20	38	/	/	/
Norm21	47	/	/	/
Norm22	43	/	/	/
Norm23	46	/	/	/
Norm24	38	/	/	/
Tumor5	53	IIA2	1	1
Tumor6	44	IB1	3	0
Tumor7	44	IB1	1	0
Tumor8	46	IB1	1	0
Tumor9	41	IIa2	1	0
Tumor10	45	IB1	2	0
Tumor11	45	IB1	2	0
Tumor12	48	IB2	1	1
Tumor13	37	IB1	1	0
Tumor14	35	IB 1	2	0

Tumor15	49	IB2	2	0
Tumor16	52	IIA1	2	0
Tumor17	43	IIA1	2	0
Tumor18	39	IIA2	3	0
Tumor19	47	IB1	2	0
Tumor20	41	IB1	1	0
Tumor21	45	IB1	1	0
Tumor22	48	IB2	2	0
Tumor23	56	IB1	2	0
Tumor24	52	IIA1	2	0
Tumor25	50	IB1	2	0
Validation phase				
N1	40	/	/	/
N2	45	/	/	/
N3	34	/	/	/
N4	37	/	/	/
N5	45	/	/	/
N6	36	/	/	/
N7	39	/	/	/
N8	48	/	/	/
N9	43	/	/	/
N10	46	/	/	/
N11	48	/	/	/
N12	53	/	/	/
N13	58	/	/	/
N14	42	/	/	/
N15	28	/	/	/
N16	32	/	/	/
N17	56	/	/	/
N18	38	/	/	/
N19	51	/	/	/
N20	49	/	/	/
N21	55	/	/	/
N22	52	/	/	/
N23	47	/	/	/
N24	35	/	/	/
N25	55	/	/	/
N26	43	/	/	/
N27	33	/	/	/
N28	39	/	/	/
N29	49	/	/	/
N30	45	/	/	/
N31	51	/	/	/

N32	38	/	/	/
N33	42	/	/	/
N34	51	/	/	/
N35	37	/	/	/
N36	39	/	/	/
N37	49	/	/	/
N38	37	/	/	/
N39	47	/	/	/
N40	35	/	/	/
N41	45	/	/	/
N42	60	/	/	/
N43	51	/	/	/
N44	33	/	/	/
N45	37	/	/	/
N46	44	/	/	/
N47	45	/	/	/
N48	41	/	/	/
N49	49	/	/	/
N50	44	/	/	/
N51	43	/	/	/
N52	55	/	/	/
N53	35	/	/	/
N54	45	/	/	/
N55	61	/	/	/
N56	38	/	/	/
N57	42	/	/	/
ADC1	41	IB1	1	0
ADC2	42	IB1	1	0
ADC3	60	IB1	1	0
ADC4	40	IIA2	2	0
ADC5	38	IB1	1	1
ADC6	34	IB1	2	0
ADC7	47	IB1	2	0
ADC8	64	IB1	3	0
ADC9	34	IB1	2	0
ADC10	53	IIA1	2	0
ADC11	59	IB1	2	0
ADC12	46	IB1	1	0
ADC13	59	IB1	2	0
ADC14	58	IB1	2	1
ADC15	51	IIA2	2	0
ADC16	54	IB1	3	1
ADC17	41	IB1	1	0

ADC18	38	IB1	1	0
ADC19	56	IB2	2	0
ADC20	51	IIA2	2	0
ADC21	42	IB1	1	0
ADC22	45	IIA1	2	0
ADC23	43	IB1	3	0
ADC24	47	IB1	3	1
ADC25	50	IIA2	2	0
ADC26	35	IIA2	2	1
ADC27	36	IB1	3	0
ADC28	48	IIA2	1	1
ADC29	49	IB1	1	0
ADC30	36	IB1	1	0
ADC31	55	IIA2	3	1
ADC32	64	IB1	1	0
ADC33	33	IB1	3	0
ADC34	35	IB1	1	0
ADC35	65	IB1	3	0
ADC36	43	IB2	3	0
ADC37	46	IB1	3	0
ADC38	55	IB1	2	0
ADC39	38	IB1	2	0
ADC40	47	IB1	2	0
ADC41	47	IB1	2	0
ADC42	53	IB1	2	0
ADC43	44	IB1	1	0
ADC44	46	IIA1	2	1
ADC45	58	IB1	3	1
ADC46	45	IB1	1	0
ADC47	57	IB1	2	1
ADC48	37	IIA1	2	1
ADC49	65	IB1	3	0
ADC50	37	IB1	2	0
ADC51	44	IIA1	3	0
ADC52	41	IB1	2	0
ADC53	63	IB1	2	1
ADC54	59	IB1	1	0
ADC55	46	IB2	1	0
ADC56	47	IB1	1	0
ADC57	27	IB1	1	0
ADC58	28	IB2	3	0
ADC59	50	IB2	3	1
ADC60	31	IB1	1	1

ADC61	50	IB1	3	0
ADC62	53	Ib2	2	1
ADC63	47	IIA2	3	0
ADC64	65	IB1	2	0
ADC65	36	IB2	2	0
ADC66	53	IB1	2	0
ADC67	38	IB1	1	1
ADC68	44	IB1	1	0
ADC69	60	IB2	2	0
ADC70	47	IB1	2	0
ADC71	48	IB1	1	0
ADC72	60	IB1	1	0
ADC73	45	IB1	3	0
ADC74	48	IB1	3	0
ADC75	52	IB1	2	0
ADC76	48	IB1	1	0
ADC77	48	IB1	3	1
ADC78	42	IB1	3	0
ADC79	43	IB1	1	0
ADC80	30	IB1	1	0
ADC81	46	IB2	2	0
ADC82	39	IB1	1	0
ADC83	58	IB1	1	0
ADC84	48	IB1	3	0
ADC85	37	IB1	1	0
ADC86	54	IB2	3	0
ADC87	59	IB1	1	0
ADC88	45	IB1	2	0
ADC89	49	IIA1	3	1
ADC90	41	IB1	1	0
ADC91	50	IIA2	1	0
ADC92	36	IB1	1	0
ADC93	40	IB1	1	0
ADC94	42	IB1	3	0
ADC95	47	IB1	2	1
ADC96	54	IB2	2	0
ADC97	60	IB2	2	0
ADC98	44	IB1	2	0
ADC99	58	IB1	1	0
ADC100	42	IB2	3	0
ADC101	52	IB1	1	0
ADC102	50	IB1	1	0
ADC103	58	IB1	1	1

ADC104	42	IIA2	2	0
ADC105	34	IB1	1	0
ADC106	61	IIA1	2	0
ADC107	36	IB1	2	0
ADC108	74	IB1	2	0
ADC109	55	IB1	2	1
ADC110	44	IB1	2	0
ADC111	43	IB1	1	0
ADC112	50	IB1	1	0
ADC113	55	IB1	1	0
ADC114	49	IB1	1	0
ADC115	39	IB1	1	0
ADC116	44	IB1	2	0
ADC117	45	IB1	2	0
ADC118	40	IB1	1	0
ADC119	42	IB1	2	0
ADC120	41	IB1	3	0
ADC121	52	IB1	2	0
ADC122	39	IB1	2	0
ADC123	41	IIA2	1	1
ADC124	43	IB2	2	1
ADC125	46	IB1	2	0
ADC126	40	IB1	2	0
ADC127	57	IB1	1	0
ADC128	33	IB1	2	1
ADC129	41	IB1	1	0
ADC130	41	IB1	3	0
ADC131	55	IB1	1	0
ADC132	44	IB1	2	0
ADC133	29	IB1	2	0
ADC134	59	IB2	2	0
ADC135	39	IB1	1	0
ADC136	41	IB1	2	0
ADC137	49	IIA1	2	1
ADC138	50	IB1	2	0
ADC139	46	IB1	2	0
ADC140	47	IB1	1	0
ADC141	54	IIA1	1	1

a, refered to FIGO 2014.

b,1, Well differentiated; 2, Moderate; 3, Poor.

c, 0, No; 1, Yes.

Table S2. Sequences of primers

Primers	Strand	Sequences（5'-3'）
ANKS4B	Forward	ACTCGTTACCACCAAGCTGC
ANKS4B	Reverse	AAGTTCCCATGGTAGGCTGC
CDH17	Forward	CCTCAGTGCACCTGGAAGTC
CDH17	Reverse	GACAGTCGGGCATGAGTACC
CLRN3	Forward	AGCATCAGCAACCCTTACCA
CLRN3	Reverse	GTTGGTTGGACTGCGTGTTC
MUC13	Forward	AAGAATGTGGAACCCGCCAT
MUC13	Reverse	CCCGGAGGCCAGATCTTTAC
SMIM24	Forward	GAAAAAGAGCCCGGAGACCA
SMIM24	Reverse	CACTGCTTTTAGGGGGCAGA
SPINK1	Forward	CCTGGCTCCTTTCACCTTTCTT
SPINK1	Reverse	GCCCCACCTACTGGGCTAT
SYT13	Forward	TGTTTGTGACTCGCCTGGAA
SYT13	Reverse	CCGCTTCTTTAGGGCTGTCT
VIL1	Forward	GCAGATATGGAGGATCGAGGC
VIL1	Reverse	CTGCTGGCTGTCTTGTGGATA
CTSE	Forward	CAGTCCAGCACATACAGCCA
CTSE	Reverse	CCACGGTTAGTCCTTCCACAG
HNF4A	Forward	CGACTCTCCAAAACCCTCGT
HNF4A	Reverse	TGATGGGGACGTGTCATTGC
MYO1A	Forward	CAGCCATAGCTGAGAGTTGGA
MYO1A	Reverse	CCCTTCATCCAGCCAGGAAA
MYO7B	Forward	CAGTAGATCTGCACAGGTGGC
MYO7B	Reverse	CACCTTCTCCAGGTTGGACAT
CAPN8	Forward	TGAAGAGCTGCTTTACCGGG
CAPN8	Reverse	AGCTGTCCATTCTTGGTGGG
RNF128	Forward	GTAGCGGAGAAGACTGGAGC
RNF128	Reverse	GGTGGGGAAGGAATGGAGTG
CEACAM5	Forward	ACACATCTTACAGATCAGGGGA
CEACAM5	Reverse	GCTGGAAAGTCCCATTGACA
FOLR1	Forward	AGTACAGATTTCTCCGGCGTG
FOLR1	Reverse	GCCCATGCAATCCTTGTCTG
SCGB1D2	Forward	GCACGGATCAGATGTCCCTT
SCGB1D2	Reverse	CTGCAGTGAAGATCAGGGTGT
SCGB2A1	Forward	CTGCAGAGGCTATGGGGAAA
SCGB2A1	Reverse	GCCAAACGCCTTGGGTAAAG
SCGB2A2	Forward	CTCTGAGCAATGTTGAGGTGT
SCGB2A2	Reverse	GAGAAGGTGTGGTTTGCAGC
LTF	Forward	TTCCCTGCTGTCGTCTTAGC
LTF	Reverse	GGAAGGGAAGGTCCAACTGT

SCNN1G	Forward	ACTACCAACTGCATCGAGCC
SCNN1G	Reverse	TTGTGCCAGGCTTGTGGTTA
HNF1A-AS1	Forward	CCTGGGCGACAGAGTGAGA
HNF1A-AS1	Reverse	TGAATGCCAGGATGAGGG
MIR194-2HG	Forward	TTCCTACACCTCGTCATCCAC
MIR194-2HG	Reverse	CAAAGAAAGACCCTGTCTCAAATA
LINC00675	Forward	CTCCTGCGAGCACATATCAA
LINC00675	Reverse	CTGGTCAGTGTCAAAGGGTAGA
LOC101060400	Forward	AGGCTGTGACTTCTGTTAGTGG
LOC101060400	Reverse	GGTGGCTTGTATTCCTTTGG
LOC105371049	Forward	GGAGGACGAGACCAACTGC
LOC105371049	Reverse	TTTCGGTGATGGGCTGAC
SSTR5-AS1	Forward	CAACATGGCTATATGGAAAGCA
SSTR5-AS1	Reverse	CTTTCTCGTTCCCGCTCA
C8orf34-AS1	Forward	ACCGATCTTGTTCTTCTCCAGG
C8orf34-AS1	Reverse	TGATGAGTGCTCCAGTCTTCAC
PPIGP1	Forward	ACATTGCCATTAACAACCTGC
PPIGP1	Reverse	TCCTCGTCCATTTCCTTCAC
KCCAT333	Forward	CAATGGTTTGATGCCAATATGT
KCCAT333	Reverse	GAAAGTGCTATCTAAGTTGGAGGT
LINC01541	Forward	ACAATCACAAGGTCCCACAAC
LINC01541	Reverse	CTTAGCCTCCCAGCCTACAT
ANAPC1P1	Forward	TTTTGGTTCATTACGGGTGC
ANAPC1P1	Reverse	CTGAGATGTGCTGTGAGGGA
HPV16 E6	Forward	CTGCAAGCAACAGTTACTGC
HPV16 E6	Reverse	GGCTTTTGACAGTTAATACACC
HPV16 E7	Forward	CATGGAGATACACCTACATTGC
HPV16 E7	Reverse	CACAACCGAAGCGTAGAGTC
HPV18 E6	Forward	ATCCAACACGGCGACCCTAC
HPV18 E6	Reverse	TTTATGGCATGCAGCATGGG
HPV18 E7	Forward	ATCCAACACGGCGACCCTAC
HPV18 E7	Reverse	CCCAGCTATGTTGTGAAATC
GAPDH	Forward	GACAGTCAGCCGCATCTTCT
GAPDH	Reverse	TTAAAAGCAGCCCTGGTGAC

Table S3. Area Under the Curve in 198 clinical samples

Test Result Variable(s)	AUC	P value	Asymptotic 95% Confidence Interval	
			Lower Bound	Upper Bound
VIL1	0.767	4.11×10^{-9}	0.704	0.831
HNF1A-AS1	0.774	1.58×10^{-9}	0.711	0.837

MIR194-2HG	0.629	0.005	0.554	0.703
SSTR5-AS1	0.449	0.259	0.362	0.535
miR-192-5p	0.862	1.66×10^{-15}	0.811	0.912
miR-194-5p	0.619	0.009	0.544	0.694
miR-192-5p/HNF1A-AS1/VIL1 panel	0.911	1.37×10^{-19}	0.872	0.950

Table S4. Area Under the Curve in 166 clinical samples

Test Result Variable(s)	AUC	P value	Asymptotic 95% Confidence Interval	
			Lower Bound	Upper Bound
TCT	0.853	8.54×10^{-14}	0.797	0.91
miR-192-5p/HNF1A-AS1/VIL1 panel	0.898	4.04×10^{-17}	0.851	0.945
TCT/miR-192-5p/HNF1A-AS1/VIL1 panel	0.964	1.06×10^{-22}	0.937	0.991
